# Influence of Sous Vide Cooking on Ground Beef Patties

**DOI:** 10.3390/foods12193664

**Published:** 2023-10-05

**Authors:** Savannah L. Douglas, Gabriela M. Bernardez-Morales, Brooks W. Nichols, Aeriel D. Belk, Tristan M. Reyes, Jase J. Ball, Jason T. Sawyer

**Affiliations:** 1 Department of Animal Sciences, Auburn University, Auburn, AL 36849, USA; sld0060@auburn.edu (S.L.D.); gzb0063@auburn.edu (G.M.B.-M.); bwn0004@auburn.edu (B.W.N.); adb0097@auburn.edu (A.D.B.); 2 Winpak Ltd., 100 Saulteaux Crescent, Winnipeg, MB R3J 3T3, Canada; tristan.reyes@winpak.com; 3 Adjunct, Department of Animal Sciences, Auburn University, Auburn, AL 36849, USA; jase.ball@zoetis.com

**Keywords:** Allo–Kramer shear force, cooked color, ground beef, sous vide cooking

## Abstract

With rising consumer demand for fast-food options, quick-service restaurants are constantly developing new menu items to attract consumers. Sous vide cookery has become popular for the in-home and fine dining consumer but has not been considered the first cooking option for quick service applications. Therefore, ground beef patties were manufactured to measure the influence of sous vide cooking time on the patty characteristics of moisture, color, and objective tenderness. Patties were randomly assigned a sous vide cooking time of 30, 60, or 90 min and then grilled to an internal temperature of 71.1 °C. Patties sous vide cooked for 30 min exhibited the greatest (*p* < 0.05) cook loss, Allo–Kramer Shear Force (AKSF) and were darker (L*) than patties sous vide cooked for 60 or 90 min. Additionally, neither internal redness, calculated spectral values of chroma, hue angle, or red-to-brown differed (*p* > 0.05) regardless of sous vide cooking time. Sous vide cooking duration prior to grilling the ground beef patties altered the moisture, color, and objective texture characteristics of ground beef patties.

## 1. Introduction

Evaluating the cookery methods of meat products is necessary to understand the changes that occur in meat quality because of cooking, as differences caused by cookery may alter consumer perceptions. Very little is known about the influence of sous vide cooking on ground meat. However, within the United States, the fast-food industry is very popular, and quick-service restaurants often face challenges to providing new items for consumers using ground meat. During the pandemic, a fast-food restaurant focused on menu creativity to enhance consumer demand for dining that incorporated sous vide cooking methods [1]. Sous vide is a cooking technique using low temperatures and longer cooking times, and has become popular in commercial applications [2]. It is estimated that by 2028, the sous vide market will reach USD 10.2 billion with an annualized growth rate of 5.3% [3]. Unlike traditional cooking methods, sous vide cooks the product in a controlled temperature water bath environment inside a heat-stable vacuum-sealed pouch [4]. In addition, sous vide allows professional chefs or home cooks to use cuts of meats considered undesirable and turn them into something that becomes a moist, tender, and flavorful dish [5]. In the fast-food industry, sous vide allows restaurants to hold meat at cooked temperatures, reducing cooking times. Sous vide cooking has several benefits: creating a uniform and desired texture, retaining a desirable color, preventing moisture or flavor losses, and prohibiting cross-contamination in storage [6,7,8]. Although sous vide can provide many desirable traits for consumers, it does have the potential to reduce consumer acceptability based on appearance [9]. 

As consumers remain focused on food sources and creating a sustainable and healthier lifestyle, a need for convenient foods without altering their preferred dietary restrictions is necessary. Restaurants that focus marketing attempts on food with clean labels have gained more attention from consumers and are driving greater marketing changes throughout the food industry [10]. Sous vide has garnered more attention as a meat processing tool able to maintain the quality, flavor, and nutritional value of meat that consumers prefer [11]. 

Raw or cooked color is a driving factor the consumer uses for determining the quality of a meat product. Sous vide cooking does not create a Maillard reaction on the surface of the meat. To counteract this cooking pitfall, chefs have roasted or fried the surface of the meat to achieve a roasted surface color while still using the long-time, low-temperature (LTLT) method [6]. In previous results using lamb meat, research concluded that roasting after sous vide cooking intensifies the Maillard reaction when compared to no additional cooking after sous vide [12]. 

Recommendations for using sous vide cookery are often based on variations in the objective tenderness ratings of lower-valued whole muscle cuts of beef or pork. However, little is known about the impact that sous vide has on ground meat characteristics. Undesirable cuts are not the only item used in sous vide cooking. This cooking method may provide the opportunity for quick-service restaurants to provide additional menu items without the cost of new equipment. Sous vide products can exhibit the desired palatability and have been reported to extend shelf life by inhibiting the growth of bacteria and lipid oxidation [4]. Using sous vide in a way to increase storage duration is possible. Heating fresh meat products at low temperatures for long times can reduce the quantity of vegetative cells [4]. Reducing microbial organisms in any quantity is well-documented to support the enhanced storing of meat products using a variety of aerobic or anaerobic packaging materials. Additional factors in the combination of sous vide cooking may include water activity, storage temperature, packaging materials, and irradiation to enhance the storage ability of meat and food products to obtain longer storage durations [4]. Currently, there are no specific guidelines or research practices for using sous vide as a cooking method for ground meat. Therefore, the objective of this study was to evaluate the influence of sous vide cooking time on the cooked characteristics of color, moisture, and objective texture in ground beef patties. 

## 2. Materials and Methods

### 2.1. Raw Materials

Six crossbred (Brangus) cattle were harvested by the Auburn University Lambert-Powell Meat Laboratory (Auburn, AL, USA). Cattle were harvested using commercial meat processing techniques for USDA humane slaughter. Carcasses were chilled at 2 °C (±1.25 °C) for 24 h prior to fabrication. After chilling, carcasses were fabricated into wholesale subprimals using fresh beef USDA institutional meat purchase specifications (IMPS) [13]. For this study fresh beef (*n* = 12) shoulder clods (IMPS 114) and (*n* = 12) chuck eye rolls (IMPS 116D) were removed, and subcutaneous fat was trimmed to not exceed 0.635 cm thick. Combined subprimals totaling 140 kg were coarse ground once through a 9.525 mm plate (SPECO 400, Shiller Park, IL, USA) using a commercial meat grinder (Model AFMG-48, The Biro Manufacturing Company, Marblehead, OH, USA). Coarse ground beef was then ground once through a 3.18 mm plate (SPECO 400, Shiller Park, IL, USA) with a bone eliminator attached (SPECO 400, Shiller Park, IL, USA). After final grinding, the ground beef was formed into 151 g patties using a food portioning machine (Hollymatic Corporation Model 54, Countryside, IL, USA). Formed patties were placed on trays lined with freezer paper (Kold-Lok KL18, Dixie Consumer Products LLC, Atlanta, GA, USA) and crust-frozen for 45 min at −22.2 °C to facilitate packaging. Crust frozen ground beef patties were packaged individually into thermoforming vacuum packaging using a Reiser roll-stock packaging machine (Optimus OL0924, Variovac, Zarrentin, Germany). A total of 225 patties were portioned, packaged, and randomly assigned to a time interval of 30, 60, or 90 min (*n* = 75/sous vide duration). Patties were sealed in a forming layer with an oxygen transmission rate of 0.8 cc/sq. m/24 h, and a non-forming layer with an oxygen transmission rate of 1.0 cc/sq. m/24 h (WINPAK Ltd., Winipeg, MB, Canada). The packaged product was stored in the absence of light at −22.2 °C (±2.1 °C) until laboratory analysis could be completed (1 week). 

### 2.2. Proximate Analysis and pH Value

Duplicate samples for proximate analysis (protein, moisture, fat, salt, and collagen) were evaluated after packaging. Analysis was conducted using a near-infrared (NIR) approved spectrophotometer (Food Scan™, FOSS Analytical A/S, Hilleroed, Denmark), and data processing was determined using ISIscan™ Software. Ultimate pH of the ground beef was measured by weighing 2 g into a plastic centrifuge tube, adding 20 mL of deionized water and homogenizing (Kinematica CH-6010, Brinkmann Instruments, Inc., Westbury, NY, USA) for 45 s. Homogenized ground beef pH was measured using a pH meter (Model-HI99163, Hanna Instruments, Woonsocket, RI, USA) equipped with a glass electrode. The pH meter was calibrated (pH 4.0 and pH 7.0) using 2-point standard buffers (Thermo Fisher Scientific, Chelmsford, MA, USA) prior to sampling (Table 1). 

### 2.3. Cookery Method and Cook Time 

At the time of cooking, patties were thawed for 12 h at 2 °C. Using a circulating temperature-control sous vide heating element (Model AN400-US00, Anova Culinary, San Francisco, CA, USA) in a 65-qt water bath, the water was heated until reaching 60 °C. Vacuum-packaged patties were placed into the water bath and submerged. Once the cooking time of 30, 60, or 90 min was complete, patties were removed from the water bath, packaging was removed, and the patty was blotted dry with a paper towel and weighed on a calibrated scale (Model PB3002-S, Mettler Toledo, Columbus, OH, USA). A commercial grill pre-heated to 148.8 °C was used to mimic an industry grilling method (Model XPE12, Garland Commercial Ranges, Mississauga, ON, Canada) following the use of sous vide. Each patty was cooked until reaching an internal temperature of 71 °C using a thermometer (Therma K-Plus, American Fork, UT, USA). The time each patty was grilled was recorded in seconds. After removal from the clamshell grill, patties were weighed again on the calibrated scale to obtain final cooked weights. 

### 2.4. Cook Loss 

Total cook loss percentage was calculated with the following: (sous vide cooked weight − grilled cooked weight) ÷ sous vide cooked weight × 100. Packaged patties were removed from packaging material after sous vide cooking time and weighed on an analytical scale (Model PB3002-S, Mettler Toledo, Columbus, OH, USA). Patties were transferred immediately to the clamshell grill until reaching an internal temperature of 71.1 °C. Grilled patties were cooled to room temperature and grilled cooked weights were recorded. 

### 2.5. Instrumental Color Measurement

Once cooled, patties were sliced horizontally through the geometric center of the patty and scanned for internal cooked color using a HunterLab MiniScan EZ colorimeter (Model 45/0 LAV, Hunter Associates Laboratory Inc., Reston, WV, USA). Before data collection, the colorimeter was calibrated using a black and white tile per the manufacturer guidelines for accuracy. Instrumental color values were determined from the mean of three readings on the internal surface of each ground beef patty using illuminant A, with an aperture of 31.8 mm, and a 10° observer to measure the lightness (L*), redness (a*) and yellowness (b*) of each ground beef patty [14]. In addition, hue angle was calculated using the following: tan − 1 (b*/a*), with a greater value indicative of the surface color shifting from red to yellow. Chroma (C*) was calculated as: √a*2 + b*2 where a larger value indicates a more vivid color. Lastly, reflectance values within the spectral range 400 to 700 nm were used to capture the surface color changes from red to brown by calculating the reflectance ratio of 630 nm:580 nm. 

### 2.6. Allo–Kramer Shear Force 

Using the 5-Blade-Allo-Kramer attachment (AKSF) with a texture analyzer (Model TA-XT Icon, Texture Technologies Corp., New York, NY, USA) the objective tenderness of each patty was measured (*n* = 225; 75/treatment). Patties were cooked and cooled according to the procedures described above. After cooling to room temperature 23.3 °C, at a load cell of 500 N and a speed of 3 mm/s, each patty was cut into a 6 × 9 cm square. Each sample was sheared once, and the maximum peak force recorded during analysis was reported in newtons (N) of shear force [14]. 

### 2.7. Statistical Analysis

Data was analyzed using the GLIMMIX model procedures of SAS (version 9.2; SAS Inst., Cary, NC, USA). Least squares means were computed for all variables. When significant (*p* ≤ 0.05) F-values were observed, least squares means were separated using pair-wise *t*-tests (PDIFF option). This experiment had a completely randomized design with each experimental unit (patty) assigned to treatment group times of 30, 60, and 90 min at random. 

## 3. Results and Discussion

### 3.1. Cook Time

A concern with using sous vide is the lack of Maillard reaction that occurs on the surface of the meat products. To simulate a commercial cooking process after sous vide, patties were cooked to their specific treatment group time (30, 60, 90 min), then patties were transferred to a clamshell grill to reach an internal temperature of 71.1 °C. The cook time after sous vide was recorded in seconds (Table 2). Expectedly, the cook time on the clamshell grill did not decrease with the increase of time in the water bath as the patties were all cooked at the same temperature before grilling occurred (*p* = 0.9868). 

Unfortunately, the documented literature regarding the cooking time of ground meat patties is limited. Limited results measuring cooking time are either presented as a standardized cooking time or end-point temperature. However, cooking time was recorded in the current study to capture an understanding of the differences that occur due to cooking methods and their subsequent influence on the final cooking time. The end-point cooked temperature can be considered a greater influence on cooking time. Previous results in a ground meat patty report that longer cooking times are necessary when the end-point temperature of the meat patty increases [15,16]. 

Overall, the grilling time of each treatment group did not change, therefore, the difference that was recorded in each characteristic may be attributed strictly to the sous vide duration and not the clamshell grilling duration. 

### 3.2. Cook Loss

Since meat consists of 75% water, there can be a significant change in the overall weight of the product after cooking, especially when using a two-step cooking method such as sous vide. Measurements were calculated based on the total amount of weight lost after sous vide and grilling. The calculated loss of moisture in the current study does not represent the moisture lost only during water bath cooking, nor the moisture loss after only grilling, cook loss was recorded as the combined moisture loss of sous vide and grilling. The cook loss after grilling decreased with the increased initial cooking time of sous vide. The cook loss in the 30 min test group was greater than the cook loss for the 60 min and 90 min (*p* = 0.0001) test groups (Table 2). The decreasing moisture loss suggests that longer cooking times of a ground meat patty in a water bath may alter the total moisture loss that occurs during the final cooking method. It is plausible that the moisture lost during sous vide cooking will be greater than moisture losses during grilling. The initial moisture loss and muscle fiber shrinking during cooking will likely not lead to the patty absorbing additional moisture during grilling. However, additional research is needed to capture the moisture loss differences that occur within each cookery method (sous vide vs. grilling) to accurately assess the total moisture losses when using any two-step cooking method. 

The characteristics of meat are often associated with cooking properties [17,18]. During the cooking process, proteins are denatured, water evaporates, and there is a loss of melted fat which leads to the reduction in cooked weight of a meat product. In previous studies, a similar weight loss to the current results was reported using conventional cooking methods in meat patties and sausages [19,20]. Likewise, the cooking loss was similar in cooked beef when using steaming after sous vide [21]. In a previous study [22], pork patties cooked for a 60 s sear time experienced greater cook loss than the control group that was strictly sous vide. Secondary cooking such as grilling creates the Maillard reaction that consumers prefer in cooked meat proteins, but additional heating and cooling can alter the moisture content, causing greater cooking losses. The current results suggest that the duration of sous vide cooking may alter the moisture retention and subsequent cooking losses that occur in either sous vide or secondary cooking such as grilling. Patties cooked for 30 min exhibited a greater total cook loss supporting the previous literature that longer cooking times using sous vide result in less cook loss. 

However, the literature concludes that in long-time, low-temperature (LTLT) cooked proteins, the cook loss increases as moisture declines with greater cooking times [16,23,24]. Water in the muscle is retained by myofibrillar proteins, however, at a temperature above 60 °C these proteins shrink and can cause an influx of water loss [25]. It is plausible that sous vide cooking creates a less evaporative environment as the product is cooked within a vacuum-sealed bag. When increasing cooking temperatures, myofibrillar proteins shrink and result in greater moisture loss [16,23,24,25]. Using an alternative cooking method such as sous vide could potentially reduce the volume of moisture losses without altering the tenderness or flavor of the meat product.

### 3.3. Instrumental Color 

Even after being cooked to 71.1 °C on the clamshell grill, each patty still appeared reddish/pink on the internal surface. This could pose an issue in the industry for consumers that will not eat a burger perceived to be “undercooked”. The b* values represent the change in yellowness. Samples exhibited higher values in the 60 and 90 min sous vide treatment time (*p* = 0.0846) when comparing the 30 min treatment time to 60 min (*p* = 0.0001) and 90 min (*p* = 0.0006). Overall, L* values were significant for this study (*p* = 0.0001). The L* values represent the amount of light detected and values displayed; the 60 min treatment group had the highest value compared to the 30 min (*p* = 0.0001) and the 90 min treatment groups (*p* = 0.3510). The a* values indicate the amount of redness with a larger value indicating a redder color. This often has a greater appeal to consumers when purchasing fresh beef but not necessarily for cooked beef. With the increase in sous vide cooking time, numerically, the a* values of the internal surface of the cooked patties increased. Regardless, the objective color measurement of redness (a*) was not different across the sous vide cooking times suggesting that the degree of doneness as perceived by consumers would not be altered for sous vide patties (Table 3). Based on objective color measurements, the internal cooked color does not differ after 30 min of sous vide cookery. Cooking ground beef patties for longer than 30 min does not improve the degree of doneness after reaching a final end-point temperature of 71 °C. 

When the cooking of a product takes place, there are several physical changes that take place as well [26]. The change in the color of a meat product is one of the most noticeable to a consumer’s eye. Much of the research and the literature relating to cooked color provides information on the surface color of a product instead of the internal color. Notably, the surface color values of a protein product cooked using sous vide can be altered and subsequently increase the browning index values as stated by previous research [27]. 

Unfortunately, the documented literature describing the sous vide implications on meat product characteristics is primarily focused on whole-muscle cuts such as steaks. The current results provide a limited understanding of the changes sous vide causes in minced meat products, but additional research is needed to elicit more information regarding cooking techniques such as sous vide. In a similar study comparing the effect of high-pressure processing (HPP) on beef steaks that were intended for sous vide, it was reported that sous vide cooking did not significantly alter the color of post-HPP beef samples except for those that were processed the longest and with the highest amount of pressure. Whole-muscle beef steaks cooked using sous vide resulted in higher a* and b* values than those in atmospheric pressure conditions [26,28]. These previous studies contrast with the current results; the longer the ground meat product is cooked using sous vide, the more the cooked color values decrease indicating a less vivid color. In reference to a previous study, it was found that meat cooked using sous vide had a more intense red color [26]. Similar results in the current study on ground meat patties resulted in higher a* values for patties cooked for the longer time increments. Due to myoglobin degradation, it is possible that the sous vide cookery in combination with packaging provides a protective mechanism for the minced meat patty which may limit the amount of myoglobin breakdown [26]. It has been suggested by previous authors that an additional cooking method should be incorporated when considering the use of sous vide to improve on the potential drawbacks of the method that may alter color, texture, or moisture [4,13]. Using sous vide as the only cooking method could decrease the consumer acceptability of a product [9]. Using additional cooking methods in combination with sous vide cooking allows for a Maillard reaction on the meat surface which plausibly could provide a more common and appealing appearance that consumers are seeking in cooked meat products. 

There was no difference observed (*p* = 0.9869) for hue angle value which indicates the shift in color from red to yellow. There was no difference observed (*p* = 0.7885) in the chroma (C*) values that represent a total measure of color (Table 4). 

In addition, spectral values were calculated from the CIE measurement of L*, a*, and b*. Color measurements were taken from cooked meat patties at three different locations after slicing patties through the geometric center. There was no interaction between any of the cooking test times and hue angle or chroma (*p* > 0.05) suggesting that sous vide does not enhance or negatively affect the internal cooked color of the patties. However, there was a significant difference in the reflectance ratio of 630 nm:580 nm and this value represents a change in color from red to brown. The 60 min patties were found to have a higher RTB value indicating that the 60 min test group had the largest amount of change from red to brown. This is evidence that the sous vide cook time does not influence the vividness or the change in color of the ground beef patties. It does, however, exhibit the change in color from red to brown. The 90 min cooking duration of ground beef patties displayed the least amount of cooked color change in red-to-brown appearance. It is plausible that the changes in red-to-brown are influenced by the grilling cook time as this treatment group required less time to reach the end-point temperature of 71 °C. 

### 3.4. Allo–Kramer Shear Force 

One of the perceived benefits to the sous vide cooking method is the potential for improving the overall tenderness of a cooked product. Objective tenderness was measured via Allo–Kramer Shear Force (AKSF) and reported in newtons (N) of force. Sheaf force values were greatest in patties sous vide cooked for 30 min compared to patties cooked for 60 min (*p* = 0.0081) or patties cooked for 90 min (*p* < 0.0001). The least amount of shear force was recorded on the patties sous vide cooked for 90 min (Table 2). As sous vide cooking time increased, the amount of Allo–Kramer force required to shear through the patty declined. 

Limitations in the literature on the objective tenderness results of meat patties cooked using a sous vide method hinders support of the current results. Interestingly, in a study using sous vide on whole-muscle pork loins cooking for varying allotments of time, reported pork loins cooked longer than 60 min resulted in a notable increase in objective firmness [16]. Obviously, whole-muscle cooked characteristics would contrast the current results reported in ground meat patties. It is likely that mincing, grinding, and forming meat patties may also contribute to altering the objective tenderness values, in addition to cooking in sous vide. Additionally, the literature states that the increase of firmness with a longer sous vide cooking time can be related to the temperature associated with the denaturation of tropocollagen at 68 °C. Cooking sous vide at anything greater than 70 °C can cause an increased cook loss [23]. Moreover, the denaturation of myofibrillar proteins and water loss has been well documented to cause variation in meat toughness [4,23]. Reported differences in these pork loins could likely be linked to the increase of cook loss. Similarly, the results of this current study show that the patties cooked the longest experienced the least amount of cook loss and had the lowest shear force values indicating that the loss of moisture in a product has a direct link to the tenderness. 

In previous research, beef muscles were ranked according to the objective tenderness values of Warner–Bratzler Shear Force (WBSF). It was found that the *triceps brachii* from the shoulder clod was considered intermediate in terms of WBSF with values of force ranging from 38.2 to 45.1 N [29]. In this current study, shoulder clods were used as part of the trimmings to form the patties. A muscle such as the *triceps brachii* may have contributed to the increases in AKSF required to shear the patties. Contrastingly, it is recognized that through the process of grinding, forming, and freezing the disadvantages associated with a tougher muscle from the beef chuck may have been eliminated. 

Previous literature suggests that sous vide-processed beef has more space between the muscle fibers in comparison to raw beef or boiled beef [2]. As the internal muscle temperature increases the internal space within meat becomes thinner causing connective tissue to dissolve allowing for more space between the muscle fibers. In summary of this previous study, sous vide samples had an increase of shrinkage which coincides with the greater loss of water previously reported [2,30].

## 4. Conclusions

Sous vide cooking time does alter the quality attributes of ground beef patties that include objective texture and internal cooked color. Patties with a longer cooking time exhibited less change in cooked color as the 90 min treatment group exhibited the highest redness value. However, cooking patties in sous vide for longer than 30 min had no effect on the internal cooked color. Additional research is needed to further identify the sensory taste impacts on ground beef using sous vide as the primary cooking method compared to other cookery methods. Furthermore, the evaluation of moisture losses using a two-step cooking method, such as sous vide and grilling ground meat patties, is necessary to improve our foundational knowledge of meat cookery. 

## Figures and Tables

**Table 1:** Proximate analysis and ultimate pH level of raw ground beef trimmings.

pH	Moisture	Protein	Fat	Collagen
5.712	66.81	22.04	18.13	3.742

**Table 2:** Influence of sous vide cook time on cook loss, grill time, and Allo–Kramer shear force.

TRAIT	30 MIN	60 MIN	90 MIN	SEM *	*p*-Value
COOK LOSS (%)	21.23 ^a^	17.33 ^b^	15.35 ^c^	0.469	0.0001
GRILL TIME (s)	38.00	37.50	37.50	2.511	0.9868
AKSF (N)	212.6 ^a^	199.6 ^b^	183.6 ^c^	3.372	0.0001

^a–c^ Mean values within a row lacking common superscripts differ (*p* < 0.05). * SEM, standard error of the mean.

**Table 3:** Influence of duration of sous vide on the internal cooked color (L*, a*, b*) values of ground beef patties.

TRAIT	30 MIN	60 MIN	90 MIN	SEM *	*p*-Value
L* ^1^	58.08 ^b^	60.41 ^a^	59.98 ^a^	0.327	0.0001
a* ^2^	15.93	16.71	16.77	0.417	0.2878
b* ^3^	18.97 ^b^	20.08 ^a^	19.97 ^a^	0.201	0.0001

^1^ L* Values are a measure of darkness to lightness (larger value indicates a lighter color); ^2^ a* values are a measure of redness (larger value indicates a redder color); and ^3^ b* values are a measure of yellowness (larger value indicates a more yellow color). ^a–b^ Mean values within a row lacking common superscripts differ (*p* < 0.05). * SEM, standard error of the mean.

**Table 4:** Calculated spectral values of the influence sous vide has on ground beef patties.

TRAIT	30 MIN	60 MIN	90 MIN	SEM *	*p*-Value
CHROMA ^1^	50.88	38.00	24.96	0.792	0.7885
HUE ANGLE (°) ^2^	50.71	38.00	26.21	3.567	0.9869
RTB ^3^	50.35 ^b^	38.50 ^a^	26.14 ^a^	0.548	0.0414

^1^ C* (Chroma) is a measure of total color (larger number indicates a more vivid color). ^2^ Hue angle (°) represents the change in color from the true red axis (larger number indicates a greater shift from red to yellow). ^3^ RTB is the reflectance ratio of 630 nm ÷ 580 nm and represents a change in the color of red to brown (larger value indicates a redder color). ^a–b^ Mean values within a row lacking common superscripts differ (*p* < 0.05). * SEM, standard error of the mean.

## Data Availability

The data used to support the findings of this study can be made available by the corresponding author upon request.

## References

[1] Restaurant Business. https://www.restaurantbusinessonline.com/food/how-arbys-spent-two-years-perfecting-its-first-burger.

[2] Chotigavin N., Kerr W.L., Klaypradit W., Kerdpiboon S. (2023). Novel sous-vide pressure technique affecting properties of local beef muscle. LWT Food Sci. Technol..

[3] Sous Vide Machine Market. https://www.adroitmarketresearch.com/industry-reports/sous-vide-machine-market.

[4] Schellekens M. (1996). New research issues in sous-vide cooking. Trends Food Sci. Technol..

[5] Baldwin D. (2012). Sous vide cooking: A review. Int. J. Gastron. Food Sci..

[6] Myhrvold N., Young C., Bilet M. (2011). Techniques and Equipment. Modernist Cuisine: The Art and Science of Cooking.

[7] Church I.J., Parsons A.L. (2000). The sensory quality of chicken and potato products prepared using cook-chill and sous vide methods. Int. J. Food Sci. Technol..

[8] Keller T., Benno J., Lee C., Rouxel S. (2008). Under Pressure: Cooking Sous Vide.

[9] Dominguez-Hernandez E., Salaseviciene A., Ertbjerg P. (2018). Low-temperature long-time cooking of meat: Eating quality and underlying mechanisms. Meat Sci..

[10] A Clean Label Challenge for Product Developers. https://www.foodprocessing.com/product-development/rd/article/11326386/a-clean-label-challenge-for-product-developers.

[11] Kato H.C.D.A., Lourenço L.D.F.H., Araújo E.A.F., Sousa C.L., Joele M.R.S., Ribeiro S.D.C.A. (2016). Change in physical and chemical characteristics related to the binomial time-temperature used in sous pasteurization. Arq. Bras. Med. Vet. Zootec..

[12] Roldan M., Loebner J., Degen J., Henle T., Antequera T., Ruiz-Carrascal J. (2015). Advanced glycation end products, physico-chemical and sensory characteristics of cooked lamb loins affected by cooking method and addition of flavour precursors. Food Chem..

[13] United States Department of Agriculture (2020). Fresh Beef Series, Institutional Meat Purchase Specifications. https://www.ams.usda.gov/sites/default/files/media/IMPS100SeriesDraft2020.pdf.

[14] American Meat Science Association (2016). Research Guidelines for Cookery, Sensory Evaluation, and Instrumental Tenderness Measurements of Meat.

[15] Liu M., Berry B. (1996). Variability in color, cooking times, and internal temperature of beef patties under controlled cooking conditions. J. Food Prot..

[16] Perez-Palacios T., Caballero D., Gonzalez-Mohino A., Mir-Bel J., Antequera T. (2019). Near Infrared Reflectance spectroscopy to analyse texture related characteristics of sous vide pork loin. J. Food Eng..

[17] Choi Y.M., Choe J.H., Cho D.K., Kim B.C. (2012). Practical use of surimi-like material made from porcine longissimus dorsi muscle for the production of low-fat pork patties. Meat Sci..

[18] Alakali J.S., Irtwangs S.V., Mzer M.T. (2010). Quality evaluation of beef patties formulated with Bambara groundnut (*Vigna subterranean* L.) seed flour. Meat Sci..

[19] Suleman R., Hui T., Wany Z., Liu H., Zhang D. (2020). Comparative analysis of charcoal grilling, infrared grilling and superheated steam roasting on the colour, textural quality and heterocyclic aromatic amines of lamb patties. Int. J. Food Sci. Technol..

[20] Naveena B.M., Khansole P.S., Kumar M., Krishnaiah N., Kulkarni V.V., Deepak S. (2017). Effect of sous vide processing on physicochemical, ultrastructural, microbial and sensory changes in vacuum packaged chicken sausages. Food Sci. Technol. Int..

[21] Modzelewska-Kapituła M., Pietrzak-Fiećko R., Tkacz K., Draszanowska A., Więk A. (2019). Influence of sous vide and steam cooking on mineral contents, fatty acid composition and tenderness of semimembranosus muscle from Holstein-Friesian bulls. Meat Sci..

[22] Cho D.K., Lee B., Hyeonbin O., Lee J.S., Kim S.Y., Choi M.C. (2020). Effect of searing process on quality characteristics and storage stability of sous-vide cooked pork patties. Foods.

[23] Roldan M., Antequera T., Martin A., Mayoral A.I., Ruiz J. (2013). Effect of different temperature-time combinations on physicochemical, microbiological, textural and structural features of sous-vide cooked lamb loins. Meat Sci..

[24] Sánchez del Pulgar J., Gázquez A., Ruiz-Carrascal J. (2012). Physico-chemical, textural and structural characteristics of sous-vide cooked pork cheeks as affected by vacuum, cooking temperature, and cooking time. Meat Sci..

[25] Christensen L., Ertbjerg P., Løje H., Risbo J., van den Berg F.W.J., Christensen M. (2013). Relationship between meat toughness and properties of connective tissue from cows and young bulls heat treated at low temperatures for prolonged times. Meat Sci..

[26] García-Segovia P., Andres-Bello A., Martínez-Monzo J. (2007). Effect of cooking method on mechanical properties, color, and structure of beef muscle (m. Pectoralis). J. Food Eng..

[27] Cho D.K., Lee B., Kim K.S., Hyeonbin O., Kim S.Y., Choi W.M. (2021). Comparison of Quality Characteristics and Palatability between sous-vide cooked pork loin patties with different searing treatments. Food Sci. Anim. Resour..

[28] Shengquin S., Sullivan G., Stratton J., Bower C., Cavender G. (2017). Effect of HPP treatment on the safety and quality of beef steak intended for sous vide cooking. Int. J. Food Sci. Technol..

[29] Sullivan G.A., Calkins C.R. (2011). Ranking beef muscles for Warner-Bratzler shear force and trained sensory panel ratings. J. Food Qual..

[30] Wattanachant S., Benjakul S., Ledward D.A. (2005). Microstructure and thermal characteristics of Thai indigenous and broiler chicken muscles. Poult. Sci..

